# A hierarchically acidity-unlocking nanoSTING stimulant enables cascaded STING activation for potent innate and adaptive antitumor immunity

**DOI:** 10.7150/thno.98272

**Published:** 2024-09-16

**Authors:** Shunyao Zhu, Tao He, Yan Wang, Yushan Ma, Wenmei Li, Songlin Gong, Yanghui Zhu, Xiangwei Wang, Xu Xu, Qinjie Wu, Changyang Gong, Yanjie You

**Affiliations:** 1 Department of Biotherapy, Cancer Center and State Key Laboratory of Biotherapy, West China Hospital, Sichuan University, Chengdu, 610041, China; 2 Department of Gastroenterology, the Third Clinical Medical College of Ningxia Medical University, Yinchuan, 750002, China; 3 Department of Gastroenterology, People's Hospital of Ningxia Hui Autonomous Region, Yinchuan, 750002, China; 4 Department of Clinical Laboratory Medicine, People's Hospital of Ningxia Hui Autonomous Region, Ningxia Medical University, Ningxia Hui Autonomous Region, Yinchuan, 750002, China; 5 School of Pharmacy, Henan University, Kaifeng, 475004, China

**Keywords:** Acidity-unlocking, neoadjuvant chemotherapy, innate immunity, adaptive immunity, liposome

## Abstract

**Rationale:** Neoadjuvant chemotherapy (NAC) has been recognized as an indispensable strategy for advanced malignancies. Nevertheless, the enhancement of overall patient survival in NAC recipients has encountered challenges due to the limited sustainability of its efficacy and the inability to prevent postoperative tumor recurrence and metastasis.

**Methods:** We devise a hierarchically unlocking nanoSTING stimulant liposome (AUG) as a neoadjuvant chemoimmunotherapy agent in the debulking of tumors prior to surgery and prevention of postoperative tumor recurrence and metastasis by simultaneously activating innate and adaptive antitumor immune responses. In the weakly acidic tumor microenvironment, the hydrazone bond within AUG is initially cleaved, leading to the release of a cyclic seven-membered ring containing tertiary amine that serve to activate the stimulator of interferon genes (STING) pathway. Following this, AUG undergoes degradation within lysosomes, facilitating the release of doxorubicin and ultimately inducing immunogenic cell death along with leakage of double-stranded DNA into the cytoplasm.

**Results:** The hierarchically acidity-unlocking pattern enables cascaded STING activation, achieving over 90% tumor growth inhibition in subcutaneous xenograft model and preventing 75% of mice from postsurgical metastasis or recurrence when combined with immune checkpoint inhibitors.

**Conclusion:** Our strategy highlights the potency of AUG as a neoadjuvant paradigm for presurgical tumor debulking and as a preventive measure against postoperative tumor recurrence and metastasis.

## Introduction

Neoadjuvant chemotherapy, also known as preoperative chemotherapy, aims to reduce tumor stage and remove circulating/metastatic tumor cells 1, 2, thereby converting inoperable tumors to operable ones, and improving patient survival 1. A considerable number of clinical trials have demonstrated the benefits of NAC in the treatment of tumors such as pancreatic cancer 3, 4, bladder cancer 5, 6, gastric cancer 7, melanoma 8, and others with improved pathologic complete remission rates (pCR). However, the overall survival rates of patients undergoing NAC have not shown significant improvement, due to the unsustainable efficacy of NAC and its inability to mitigate postoperative recurrence and metastasis resulting from residual or circulating tumor cells 9-11. Therefore, there is an urgent need to develop new neoadjuvant therapeutic strategies that can effectively promote long-term survival benefits for patients while tumor downstaging.

​Tumor immunotherapy is designed to boost natural defenses to eliminate malignant cells, either by exerting immunostimulatory effects or blocking immunosuppressive pathways 12, 13, and therefore has the potential to improve the effectiveness of NAC and increase the long-term survival of patients 14. In clinical studies, the addition of nivolumab 15, trastuzumab 16, 17 and pembrolizumab 18 to standard NAC resulted in an increased pCR compared to NAC alone, but did not significantly improve overall survival, which suggests long-term survival benefits are difficult to achieve by activating exhausted T cells alone. The initiation and sustenance of the T-cell response is contingent upon innate immune cells, which are crucial in acquiring tumor antigen information and eliciting robust adaptive anti-tumor immunity 19. Notably, in the realm of spontaneous natural antitumor T cell responses, the factors and mechanisms necessary to stimulate innate immune sensing may vary 20, 21. This represents a significant gap in our understanding, as the ability to trigger innate immune activation and subsequently elicit adaptive antitumor immune responses is crucial for enhancing the efficacy of current neoadjuvant therapies and increasing the number of patients who derive clinical benefit.

The stimulator of interferon gene (STING), a cytoplasmic DNA sensor localized to the endoplasmic reticulum (ER), plays a crucial role in the immune system by providing numerous benefits and exhibiting potent immune activation capabilities 22, 23. The cyclic GMP-AMP synthase (cGAS) / STING pathway is integral to the innate response in tumor immunity 24, 25, contributing significantly to dendritic cell maturation and facilitating cytotoxic T cell infiltration and activation 21. Therefore, STING-targeted therapy has become a new option for antitumor immunotherapy, among which ADU-S100 (MIW815) (NCT02675439) 26, MK-1454 (NCT03010176) 27 and E7766 (NCT04144140) 28 have been successively approved for clinical trials. ​However, as cytosolic sensors, the systematic delivery of STING agonists to tumors has been limited by unfavorable pharmacological properties and targeting inefficiencies due to rapid clearance in the tumor microenvironment.

**Figure 1:**
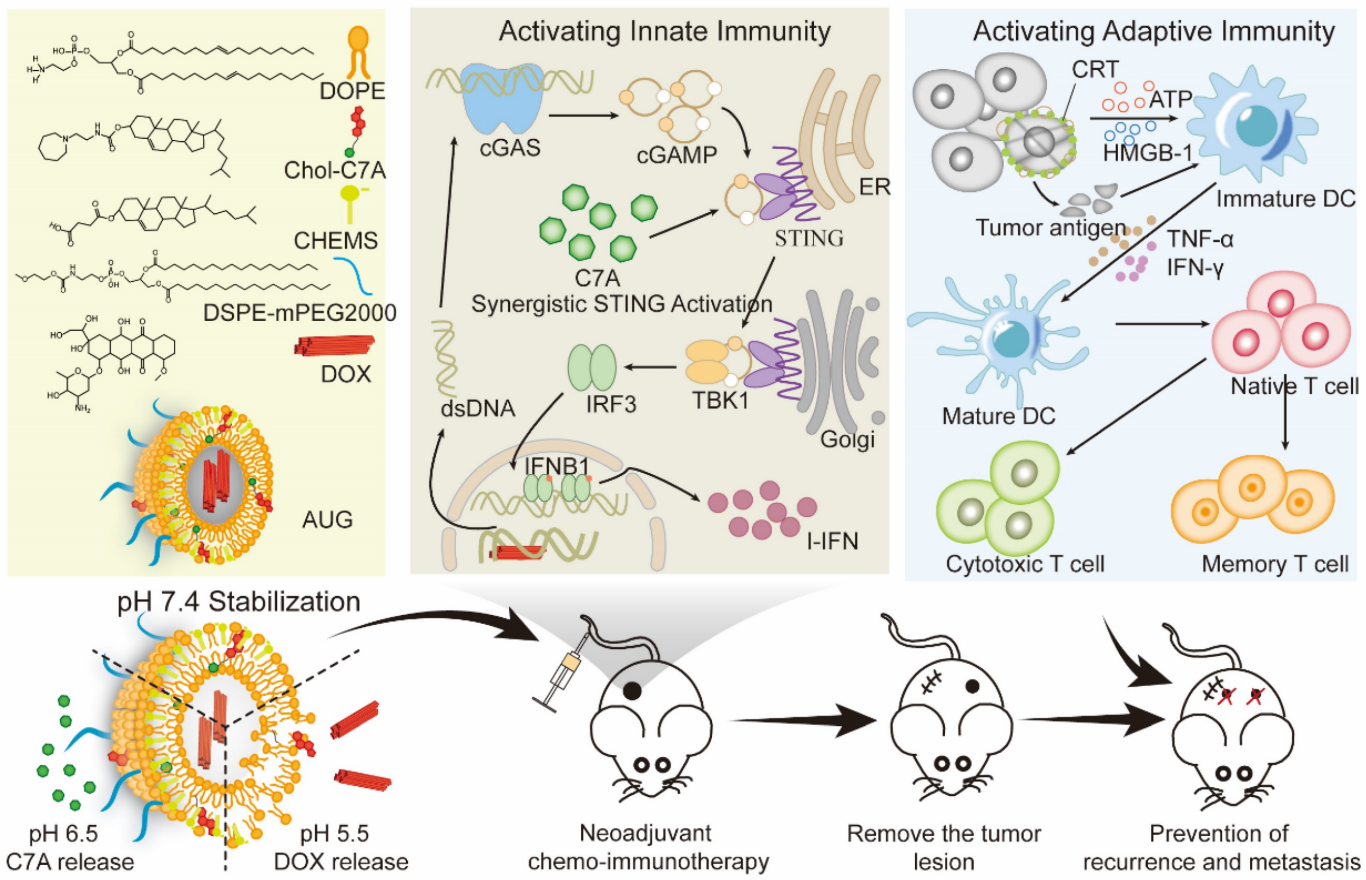
Schematic illustration of hierarchical acid-unlocking neoadjuvant chemotherapeutic agent AUG via cascaded STING activation for co-activation of innate and adaptive antitumor immunity.

Thus, we presented a hierarchically unlocking nanoSTING stimulant liposome as a neoadjuvant chemo-immunotherapy agent capable of inducing robust innate and adaptive antitumor immune responses to inhibit postoperative tumor metastasis and recurrence (Figure 1). AUG was prepared by co-encapsulating doxorubicin (DOX) and hydrazone bond coupled cholesterol-C7A (CHOL-C7A) in a pH-sensitive liposome. After system administration, AUG would be localized to the tumor site through the enhanced permeability and retention effect. Within the weakly acidic tumor extracellular tumor environment (pH 6.5), the hydrazone bond was firstly cleaved and resulting in the release of C7A, whose seven-membered tertiary amine group can directly bind to STING protein, thereby activating the immune response 29. Then, AUG was further disassembled in tumor cells under a lysosome pH (pH 5.5), achieving the intracellular release of DOX, which can induce immunogenic cell death (ICD) and subsequent release of damage-associated molecular patterns and tumor-associated antigens 30. More importantly, the topoisomerase inhibitory property of DOX hinders DNA replication, allowing dsDNA leakage into the cytoplasm 31, thus stimulating activation of the cGAS-STING pathway in conjunction with C7A, and then upregulating the expression of IFN-β, which promotes DC maturation and induces potent innate and adaptive antitumor immunity before surgery. Thus, AUG can effectively bridge chemotherapy and antitumor immunity to achieve preoperative tumor downstaging and postoperative long-term survival benefit. Our rational hierarchically unlocking strategy may pioneer a novel avenue in tumor neoadjuvant chemo-immunotherapy.

## Materials and methods

### Materials

Dioleoylphosphatidylethanolamine (DOPE), cholesteryl hemisuccinate (CHEMS), cholesterol (CHOL) and other lipids were purchased from AVT (Shanghai) Pharmaceutical Tech Co., Ltd. 2-(2-bromoethyl) isoindoline-1,3-dione, azepane, hydrazinium hydroxide solution, 2-bromo-1, 1-dimethoxyethane, sodium hydride and N-ethyl-N-isopropylpropan-2-amine were gained from Macklin. Ethanol (EtOH), tetrahydrofuran (THF), methanol (MeOH), ethyl acetate (EtOAC), hydrochloric acid, sodium hydroxide, dichloromethane (DCM), N, N-Dimethylformamide (DMF), N, N-diisopropylethylamine (DIEA) and K_2_CO_3_ that have not been specifically stated were purchased from Aladdin. Fetal bovine serum and antibiotics were gained from Gibco. STING, p-STING, TBK1 and other cGAS-STING pathway related antibodies were acquired from Cell Signaling Technology, and all other antibodies were purchased from BioLegend. The detailed information was displayed in table S1.

### Cells and animal experiments

B16-F10 cells were obtained from American Type Culture Collection (USA), and incubated in DMEM medium containing 10% fetal bovine serum and 1% penicillin/streptomycin. To generate bone marrow-derived dendritic cells (BMDCs) 32, bone marrow was extracted from female C57BL/6 mice, and cultured in RPMI 1640 complete medium supplemented with 10 ng/mL GM-CSF and 20 ng/mL IL-4. After 3 days, the medium was refreshed, with half replaced by fresh medium on day 6. BMDCs were collected on day 9. All animal experiments were purchased from Weitong LiHua Experimental Animal Technology Co. Ltd. (Beijing, China). All animal experiments were performed in accordance with the rules of the Institutional Animal Care and Treatment Committee of Sichuan University.

### Synthesis and characterization of CHOL-C7A

Amine (1.0 g) and N-(2-bromoethyl) phthalimide (2.0 g) were dissolved in acetonitrile, and K_2_CO_3_ was added as catalyst. The resulting mixture was degassed and placed in the N_2_ atmosphere via balloon and refluxed overnight. Saturated sodium bicarbonate solution was subsequently introduced, followed by extraction. The mixed organic layers were then acidified with 2 N hydrochloric acid, and water was employed for rinsing. Following pH adjustment to 12 with 4 N sodium hydroxide, methylene chloride was utilized for extracting. The resulting organic solution was dried, evaporated, and purified by flash column chromatography to give a yellow solid compound of 2-(2-(azepan-1-yl) isoindoline-1,3-dione. The obtained compound (200 mg) and hydrazine hydrate (70 mg) were dissolved in ethanol. The reaction was refluxed, and the resulting precipitate was removed by filtration to obtain C7A-NH_2_.

CHOL (1.0 g) and 4-nitrophenyl carbonochloridate (0.6 g) were dissolved in methylene chloride, and N, N-dimethylpridin-4-amine and triethylamine were added as catalysts. After stirring overnight at room temperature, the mixture is concentrated under reduced pressure to obtain CHOL-COOR.

To obtain CHOL-C7A, C7A-NH_2_ (80 mg) and CHOL-COOR (465 mg) were dissolved in a mixture of anhydrous DCM and DMF, and DIEA was added as catalysts. The mixture was stirred overnight, and then the CHOL-C7A was obtained by extraction, concentration and purification. Then, CHOL-C7A was characterized via^ 1^H-NMR and mass spectrometry.

### Preparation of pH-sensitive liposomes

AUG was prepared using the classical method of a transmembrane ammonium sulfate gradient 33. Briefly, DOPE, CHEMS, CHOL-C7A and DSPE-mPEG2000 at a molar ratio of 7:3:5.25:0.5 were dissolved and evaporated to form a lipid film 34. Then, the film was hydrated with ammonium sulfate solution (300 mM), and followed by extruded through the polycarbonate filter. AUG was obtained through incubating the concentrated liposomes with DOX in a shaker for 3 h. The DOX-Lipo was prepared utilizing an equimolar ratio of lipids, with the substitution of CHOL for CHOL-C7A 29. The C7A-Lipo was prepared using an equimolar ratio of lipids, with the substitution of PBS for ammonium sulfate solution.

### Liposome characterization

The liposomes were characterized by measuring their size distribution and zeta potential with a Malvern spray analyzer. Prior to observation via transmission electron microscopy, the prepared liposomes were negatively stained with a 2% (wt/wt) phosphotungstic acid solution. The quantification of DOX and C7A within the liposomes was performed using ultraviolet-visible (UV-vis) spectrophotometry. To assess the encapsulation efficiency (EE) of DOX, AUG were suspended in PBS and then diluted 10-fold with methanol. EE was quantified using prescribed equations:


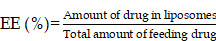
 × 100%

Moreover, the physicochemical stability of AUG, as indicated by particle size, zeta-potential, and EE, was evaluated in PBS (pH 7.4) at 4℃ over a period of one month.

### *In vitro* drug release

AUG was enclosed in dialysis bags and placed in 10 mL of PBS buffer (pH 7.4, 6.5, or 5.5) with gentle agitation (37 °C, 120 rpm). At predetermined time points (1, 2, 4, 8, 12, 24, 48, and 72 h), 0.5 mL of the PBS was sampled and replenished with an equal volume of fresh buffer. The release of DOX and C7A from liposomes was quantified using a UV-vis spectrophotometer, and the percentage of released drugs was calculated as:


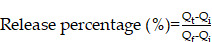
 × 100%

Where Q_f_ was the total DOX or C7A in liposomes, while Q_t_ and Q_i_ meant the initial and intermediary absorbance of the liposome suspension, respectively.

### *In vitro* cell cytotoxicity assay

The cytotoxicity of liposomes was evaluated through the utilization of the 3-(4,5-dimethylthiazol-1-yl)-2,5-diphenyltetrazolium bromide (MTT) assay. B16-F10 cells were seeded in triplicate and allowed to incubate overnight before being exposed to solutions of varying pH containing a specific concentration of liposomes. Following an additional 24 h incubation period, 10 μL of MTT solution was introduced to the cells, which were then subjected to a further 4 h incubation. Subsequent to this, the culture medium was aspirated and the formazan precipitate was dissolved in dimethyl sulfoxide. The cell viability was calculated with the absorbance at 570 nm by a microplate reader.

### Detection of dsDNA release in cytoplasm

B16-F10 cells were initially cultured in a 35-mm dish. 12 h later, the cells were incubated with PBS, free DOX, DOX-Lipo, C7A-lipo and AUG, respectively (C7A: 20 ng/mL, DOX: 40 ng/mL). Following an additional 24 h incubation period, the cells underwent treatment with 4% paraformaldehyde, 0.3% Triton X-100, and 5% BSA for permeabilization and blocking, followed by incubation with a dsDNA marker (1:100) overnight and staining with an Alexa Fluor 647 labeled secondary antibody. Detection of γ-H2AX involved permeabilization, blocking, and staining with an anti-γH2AX antibody (1:100) overnight, followed by staining with an Alexa Fluor 647 labeled secondary antibody. The fluorescence was then visualized using a confocal laser scanning microscope.

### Western blot analysis

B16-F10 cells were treated with different formulations as detailed above. Following incubation, the cells were extracted and subjected to lysis in lysis buffer for a duration of thirty minutes. Subsequently, the protein content was quantified and separated via an 8-15% SDS-PAGE gel. Following a 2 h blocking procedure with 5% skim milk, the samples were then exposed to antibodies against STING (1:1000), phospho-STING (1:1000), IRF3 (1:1000), phospho-IRF3-S396 (1:1000), TBK1 (1:1000), phospho-TBK1 (1:1000) and GAPDH (1:1000). All antibodies were purchased from CST. The protein was examined with ECL Regent after incubation with HRP-conjugated goat anti-rabbit or HRP-conjugated goat anti-mouse secondary antibody.

### Analysis of AUG-induced ICD

To investigate the impact of AUG-induced ICD on tumor cells, 2×10^5^ B16-F10 cells were seeded and treated with PBS, free DOX, DOX-lipo, C7A-lipo, or AUG for a duration of 24 h. Following treatment, the cells were subjected to immunofluorescence staining for CRT using a CRT antibody and Alexa Fluor 647 labeled secondary antibody, as well as DAPI. Subsequently, HMGB1 detection was performed by incubating the fixed and permeabilized cells with an HMGB1 antibody and staining with Alexa Fluor 647 labeled secondary antibody and DAPI. Additionally, ATP levels in the supernatant were quantified using the ATP detection kit.

### Assay of immune activation *in vitro*

B16-F10 cells were cultured in 6-well plates and treated with PBS, free DOX, DOX-Lipo, C7A-lipo, or AUG for 24 h (C7A: 20 ng/mL, DOX: 40 ng/mL). Subsequently, BMDCs were co-cultured with these treated cells for an additional 24 h. BMDCs were harvested, washed and stained by fluorophore-labeled CD11c, CD80, and CD86 antibodies before analyzed using flow cytometry.

### Biodistribution of liposomes *in vivo*

To examine the localization of liposomes following systemic administration, DiD was employed for the labeling of liposomes. The B16-F10 subcutaneous xenograft model was established. Once the tumors reached a volume of approximately 300 mm^3^, the mice received an intravenous injection of DiD labeled liposomes at a dosage of 30 μg per mouse. Subsequently, the hearts, livers, spleens, lungs, kidneys, and tumors of the mice were excised and measured under identical conditions.

### *In vivo* synergetic therapeutic efficacy by AUG

A suspension of B16-F10 cells were injected into the right back of C57BL/6 mice. When the tumors reached about 50 mm^3^, mice were divided into five groups and were injected intravenously with PBS, Free DOX, DOX-Lipo, C7A-Lipo and AUG, respectively (DOX: 4 mg/kg, C7A: 1 mg/kg). These injections were administered every 3 days for a total of 3 doses. The length and width of tumor were measured every other day using a digital caliper. Mice were considered deceased if they exhibited signs of death or if the tumor volume exceeded 2000 mm^3^.

In order to assess the therapeutic impact on postoperative metastasis, mice were randomly allocated into three groups upon reaching a tumor volume of 50-100 mm^3^, and were intravenously administered with PBS, AUG, or AUG plus αPD-1. The treatment was conducted every 3 days for 3 times. Then, tumors located in the left flank of mice were surgically excised, and the resulting wounds were closed using 5-0 silk sutures. Following surgical procedures, tumor cells were introduced into the contralateral side of each mouse the day after. The growth of contralateral tumors was observed every other day. At Day 19 post-inoculation, the mice were euthanized in a humane manner, and spleens were harvested to identify T cells expressing specific markers. A comparable study was conducted to investigate melanoma postsurgical recurrence in a murine model, wherein mice remained tumor-free for a period of 10 days following surgery before being subjected to rechallenge. Subsequently, on Day 19 post-rechallenge, the splenic tissues in mice were extracted for the purpose of identifying effector memory T cells (T_EM_) and central memory T cells (T_CM_) 35.

### *In vivo* immune response activation

The B16-F10 subcutaneous xenograft model was established as described above, and mice were treated with PBS, Free DOX, DOX-Lipo, C7A-Lipo and AUG, respectively. Tumor tissues and spleens were collected on day 2 following the final treatment and processed into single-cell suspensions. Erythrocytes were lysed, and the suspensions were filtered through a nylon mesh filter before being stained with antibodies and analyzed using flow cytometry: (i) T cells (CD3^+^CD4^+^CD8^-^ or CD3^+^CD4^-^CD8^+^), (ii) CTLs (CD3^+^CD8^+^CD69^+^), (iii) Tregs (CD4^+^CD25^+^Foxp3^+^), (iv) DCs (CD11b^+^CD80^+^CD86^+^) and (v) NK cells (CD3^-^NK1.1^+^).

To quantify the population of DCs and NK cells within tumor tissues, the tumor tissue was precisely weighted and homogenized to create a single-cell suspension. Then, erythrocytes were lysed and adjusted the cell suspension into a standardized density. Took the same volume from the regulated cell suspension and performed immunofluorescent staining with DC and NK cell markers-specific antibodies. Following staining, an equal volume was selected for testing.

### *In vivo* toxicity analysis

Blood in mice treated with different formulations as described above were collected for total protein (TP), albumin (ALB), aspartate aminotransferase (AST), alkaline phosphatase (ALP), creatine kinase-MB (CKMB), urea (UREA), white blood cell (WBC), red blood cell (RBC), hemoglobin (HGB) and platelet (PLT) measurement according to the manufacturer's instruction. In addition, the hearts, livers, spleens, lungs, and kidneys of the mice were isolated for histological examination using hematoxylin and eosin (H&E) staining.

### Statistical analyses

The data for all measurements were reported as mean ± standard deviation (SD). The mean differences were analyzed using Student's t-test or one-way analysis of variance (ANOVA). To compare all groups, a two-way ANOVA with the Bonferroni test was performed. Statistical significance was denoted as *P < 0.05, **P < 0.01, *** *P* < 0.001.

## Results and Discussion

### Preparation and characterization of AUG

**Figure 2:**
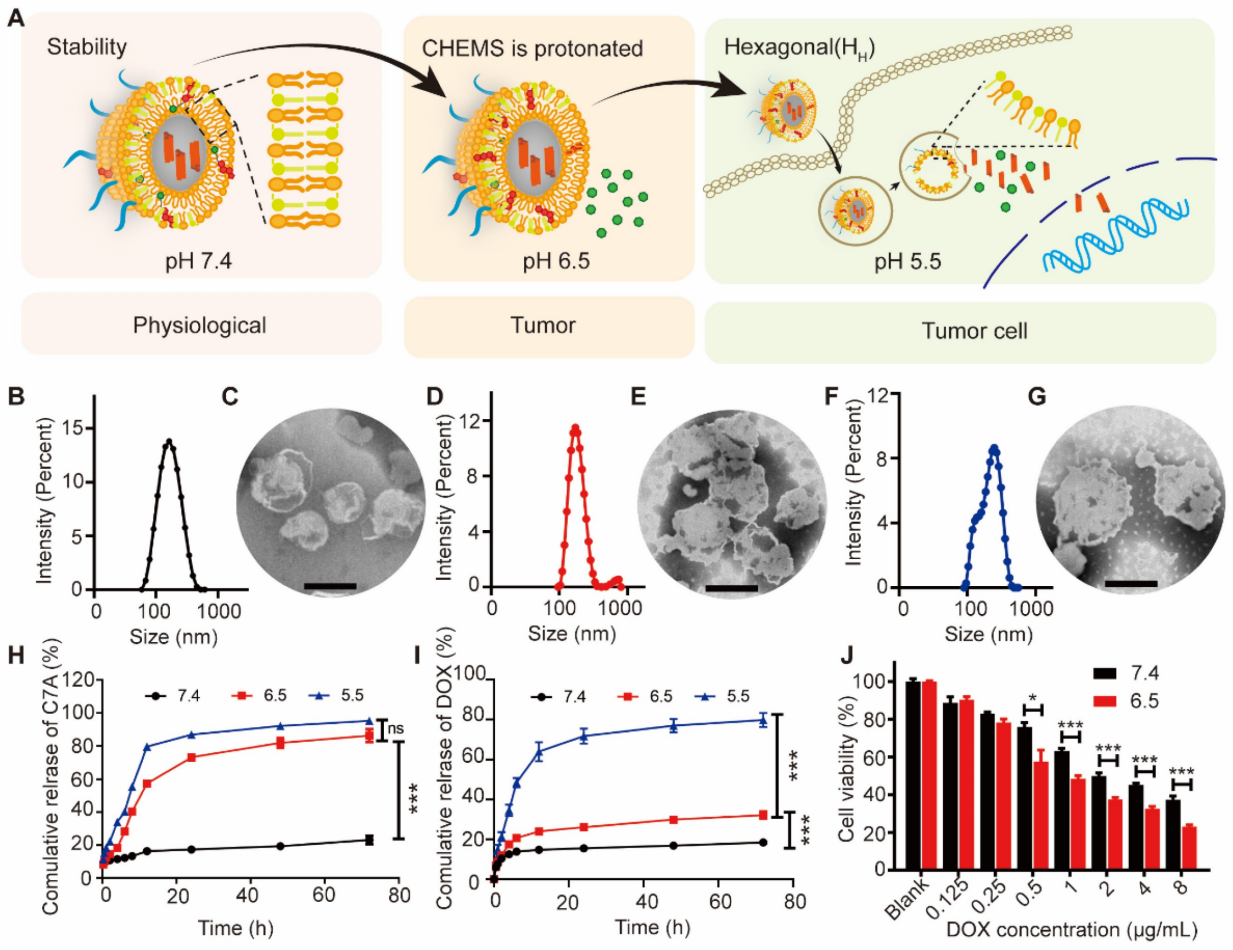
Characterization and cytotoxicity of AUG. (A) Schematic illustration of the hierarchical unlocking of the AUG system. (B and C) The particle size and visualization of AUG in pH 7.4 (Scale bar: 100 nm). (D and E) The particle size and visualization of AUG in pH 6.5 (Scale bar: 100 nm). (F and G) The particle size and visualization of AUG at RT in pH 5.5 (Scale bar: 100 nm). (H and I) *In vitro* cumulative release behaviors of C7A (H) and DOX (I) released from AUG in differential pH PBS. (J) Cell viability of cells following incubation with AUG at different concentrations of DOX under specified pH conditions. *P < 0.05, **P < 0.01, ***P < 0.001.

CHOL-C7A was synthesized via aminolysis, hydrazine hydrolysis, esterification, and ester aminolysis reactions (Figure S1, S3 and S5). All synthesized compounds were confirmed by ^1^H NMR spectra (Figure S2, S4 and S6). The exact molecular weight of CHOL-C7A was also verified by high-resolution mass spectrometry (Figure S7) ([M-H] was 554.4811). Together, these results demonstrated that CHOL-C7A has been synthesized successfully. Then, C7A-Lipo was prepared, and has a particle size of approximately 154.6 ± 1.8 nm (Figure S8). AUG were prepared using the lipid mixture of CHOL-C7A, DOPE, CHEMS and DSPE-mPEG2000 and followed with the encapsulation of DOX. The ionization-labile of DOPE and CHEMS was designed to produce acid-sensitive liposomes that could undergo charge conversion in the acidic tumor environment 32. The formation of a stable lamellar phase at physiological pH is facilitated by the presence of the stabilizing component (CHEMS) with a negatively charged functional group, which interacts with the phosphate group of DOPE to generate repulsive forces 36. However, the excess of protons lead to the neutralization of CHEMS and cause DOPE molecules to revert to their inverted hexagonal structures, thereby making the acidified AUG unstable (Figure 2A). The acid-sensitive liposomes were confirmed by measuring their particle sizes. As shown in Figure 2B, D and F, an increase in the particle sizes from 148 ± 3 nm (pH 7.4) to 160 ± 7 nm (pH 6.5) and 183 ± 9 nm (pH 5.5) was observed, respectively. Coincidental with particle size results, TEM images also displayed that the morphology of the AUG increased with the decrease of pH (Figure 2C, E and G), indicating the successful preparation of acid-sensitive liposomes. To evaluate the stability, the size and zeta potential of AUG as well as its DOX content were measured when store at 4 ℃, and results indicated that the AUG was stable up to 28 days (Table S2), and the DOX content showed low leakage in neutral conditions at 4℃.

Subsequently, the drug release kinetics were evaluated under various pH conditions simulating both the blood and tumor microenvironments. For the release of C7A, there is no significantly different in acidic environments below 6.5 due to the breakage of its connection with cholesterol in a weak acid (pH 6.5) condition (Figure 2H and S9). For the DOX, the cleavage of the CHOL-C7A bond at a pH of 6.5 induces structural instability within the AUG liposomes, leading to a controlled leakage of DOX. Despite this, the leakage remains within a confined threshold, not exceeding 30% of the encapsulated DOX. However, DOX was rapidly released at pH 5.5 due to a transition in the crystalline phase of the liposome (Figure 2I). Then, the cytotoxicity tested against B16-F10 cells demonstrated superior tumor-killing efficacy in an acidic microenvironment compared to AUG at physiological conditions (Figure 2J). One possible reason is that AUG remains stable under physiological conditions while exhibiting rapid release behavior in the acidic tumor microenvironment, thus showing stronger cytotoxicity under acidic conditions. Our results show that AUG is able to respond to different pH conditions of the tumor microenvironment and lysosomes, and achieve cascaded differential release of C7A and DOX.

### *In vitro* innate and adaptive antitumor immunity activation

**Figure 3:**
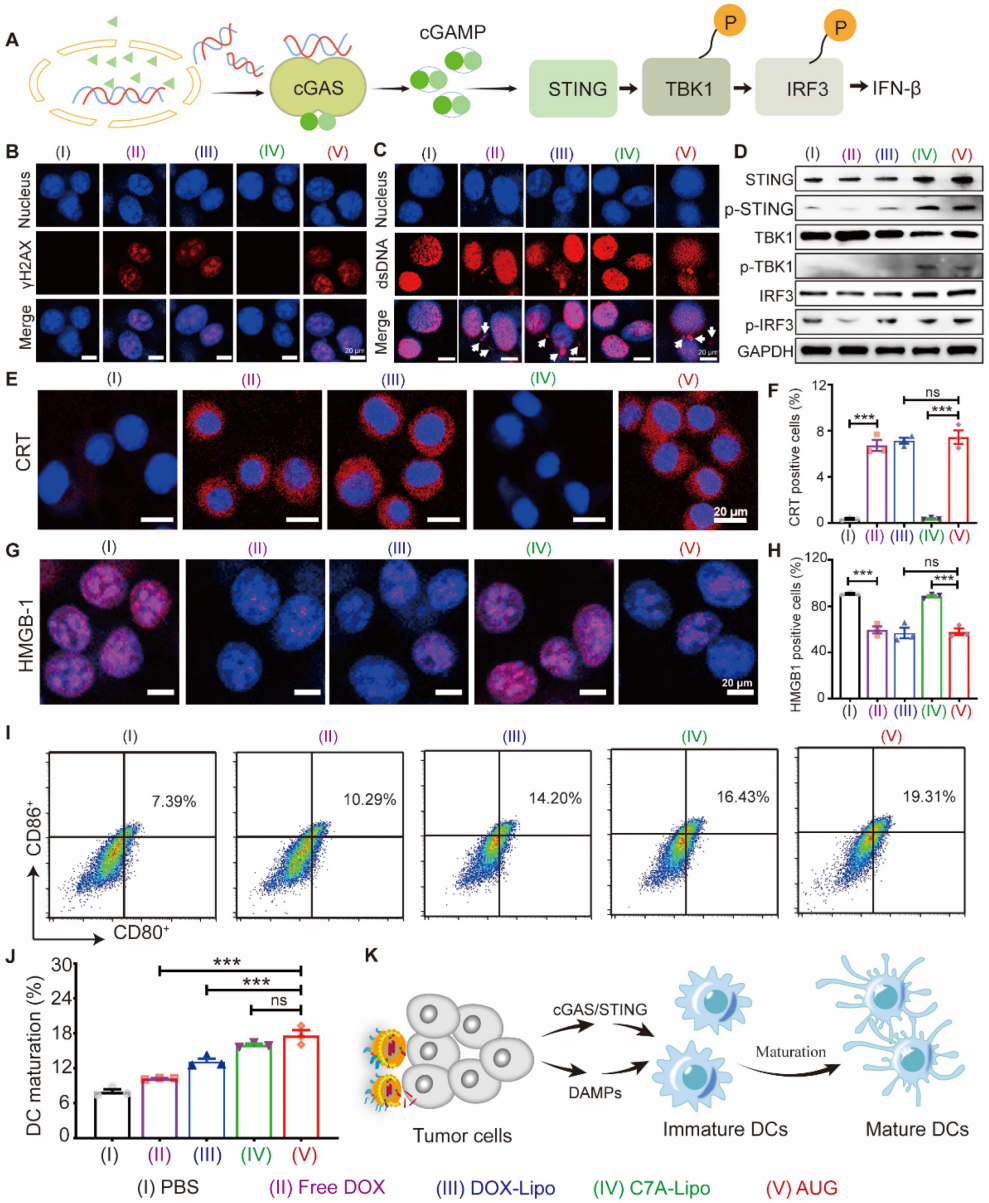
*In vitro* innate and adaptive antitumor immunity co-activation. (A) The activation of cGAS-STING pathway in the cytoplasm is depicted diagrammatically in response to the release of dsDNA. (B) Immunofluorescence images of B16-F10 cells following treatment with various formulations: nucleus (blue) and γH2AX (red). Scale bar: 20 μm. (C) Immunofluorescence of B16-F10 cells following treatment with various formulations: nucleus (blue) and dsDNA (red). Scale bar: 20 μm. (D) Expression levels of STING, p-STING, TBK1, p-TBK1, IRF3 and p-IFR3 in B16-F10 cells that were treated with different formulations. CLSM images of CRT (E) and HMGB1(G) in tumor cells treated by various formulations with a scale bar of 20 μm. The quantification of positive CRT cells (F) and positive HMGB1 cells (H) using flow cytometry. Flow cytometry analysis (I) and quantitative evaluation (J) of BMDC maturation following various treatments. (K) AUG-induced cytoplasmic DNA and ICD of tumor cells activate both innate and adaptive immunity.

The destruction of DNA via DOX in B16-F10 tumor cells may promote the activation of the cGAS-STING pathway (Figure 3A). As a proof of concept. we first stained B16-F10 cells with γH2AX, a marker of DNA double-strand breaks, and results showed that the DOX, DOX-Lipo, and AUG groups showed a distinct red fluorescence in the nucleus, indicating that formulations containing DOX caused significant DNA damage (Figure 3B). Meanwhile, significantly higher cytoplasmic dsDNA abundance was found in DOX-treated cells, but not in C7A-treated cells, demonstrating that DOX-induced nuclear DNA damage can release dsDNA into the cytoplasm (Figure 3C).

Considering that cytoplasmic dsDNA can activate the cGAS-STING pathway, which might be enhanced by C7A. We then investigated the ability of AUG to activate the cGAS-STING pathway via western blotting assay. As shown in Figures 3D and S10, DOX-Lipo up-regulated the expression of p-STING, while C7A-Lipo can directly up-regulate the expression of STING, p-STING, and p-TBK1. In contrast, AUG significantly enhanced the expression of STING, p-STING, p-IRF3, and p-TBK1 in B16-F10 cells, mainly due to the dual effects of DOX-induced dsDNA release and C7A as a STING agonist. These data suggested that the released dsDNA in the cytoplasm activated the cGAS/STING pathway, which could be further enhanced by C7A.

Chemotherapy-mediated ICD is an emerging concept in immunotherapy that can improve immunotherapy outcomes by restoring the immunogenicity of tumor cells through CRT exposure, HMGB1 and ATP release 27, 37. Our results showed that formulations containing DOX-treated B16-F10 cells presented a significantly enhanced CRT (red fluorescence) exposure compared with the PBS and C7A-Lipo groups, and the results were also supported by the flow cytometry data (Figure 3E and F). AUG-induced release of HMGB1 and ATP from B16-F10 cells was also confirmed in our results (Figure 3G, H, and Figure S11). To verify whether immunogenic dead tumor cells can initiate adaptive anti-tumor immune responses, we co-incubated different preparations treated tumor cells with BMDCs to evaluate the DC maturation. As shown in Figure 3I and J, the frequency of CD80^+^CD86^+^ BMDCs in the AUG group was obviously increased, compared with other groups. Mature DCs could interact with T-cells and drive immune response. Thus, DOX-induced cytoplasmic DNA release and ICD-induced DMAPs release may synergistically promote DC maturation to activate T cells, which contributes to the co-activation of innate and adaptive immune responses. (Figure 3K).

### *In vivo* antitumor effects of AUG

**Figure 4:**
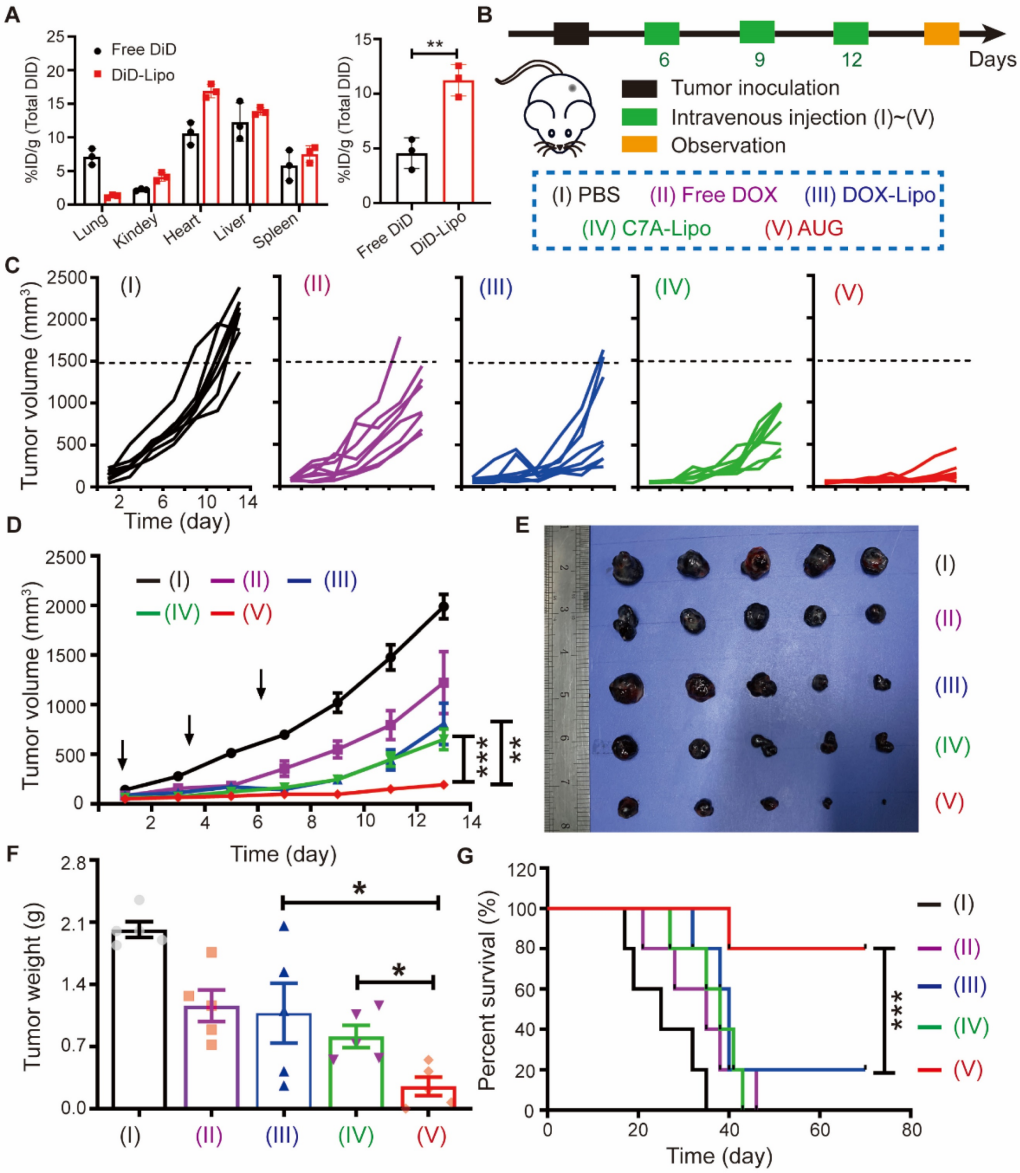
The antitumor effect of AUG. (A) The distribution of total DiD in B16-F10 bearing mice 24 h post-treatment, left: major organs; right: tumor tissue. (B) The schedule for tumor inoculation and AUG administration. (C) Tumor growth curves for each mouse. (D) Total tumor growth curves of mice in different groups post-treatment and the black arrow specifically denotes the time point of animal administration. (E and F) Photographs of dissected tumors (E) and average tumor weights (F) at the end of the treatment. (G) Survival curve of mice following different treatments. *P < 0.05, **P < 0.01, ***P < 0.001.

Before evaluating the antitumor efficacy, AUG tagged with DiD was utilized to study its biodistribution *in vivo* tumor model. As displayed in Figure 4A and S12, DiD-Lipo showed more tumor accumulation than DiD group within 24 h of administration, which was attributed to the long-circulating properties of PEG and the enhanced penetration effect of this acidic-sensitive liposome. Driven by the impressive tumor accumulation ability of AUG, we developed a subcutaneous tumor model to evaluate the anticancer efficacy (Figure 4B). Mice were administered intravenously with different formulations every 3 days for a total of 3 doses. Results depicted in Figure 4C and D indicated that DOX-Lipo exhibited a greater inhibitory effect on tumor growth compared to free DOX, which might be attributed to the enhanced tumor accumulation of acidic-sensitive liposomes. Otherwise, C7A-Lipo showed tumor inhibitory effects similar to DOX-Lipo, which might be attributed to the activation of the STING pathway by C7A. Among all groups, AUG treatment presented the strongest tumor growth inhibition (more than 90%), which might be ascribed to the co-activation of innate and adaptive antitumor immunity. *Ex vivo* tumor images and weights were also verified for the AUG-induced tumor suppression (Figure 4E and F). It was observed that the administration of AUG resulted in a significant extension of mice survival, exceeding 70 days, in comparison to alternative treatments such as PBS (25 days), free DOX (35 days), DOX-Lipo (38 days), and C7A-lipo (40 days), as illustrated in Figure 4G. Moreover, there were no discernible alterations in the body weights of the mice throughout the treatment period, as indicated in Figure S13. In addition, comparable outcomes were consistently observed within the MC38 tumor model (Figure S14), corroborating the replicability of the antitumor effects attributed to AUG. These findings imply that AUG exhibits remarkable antitumor properties and holds promise as a potent neoadjuvant intervention for the suppression of primary tumors prior to surgery.

### Antitumor immune activation by AUG* in vivo*

**Figure 5:**
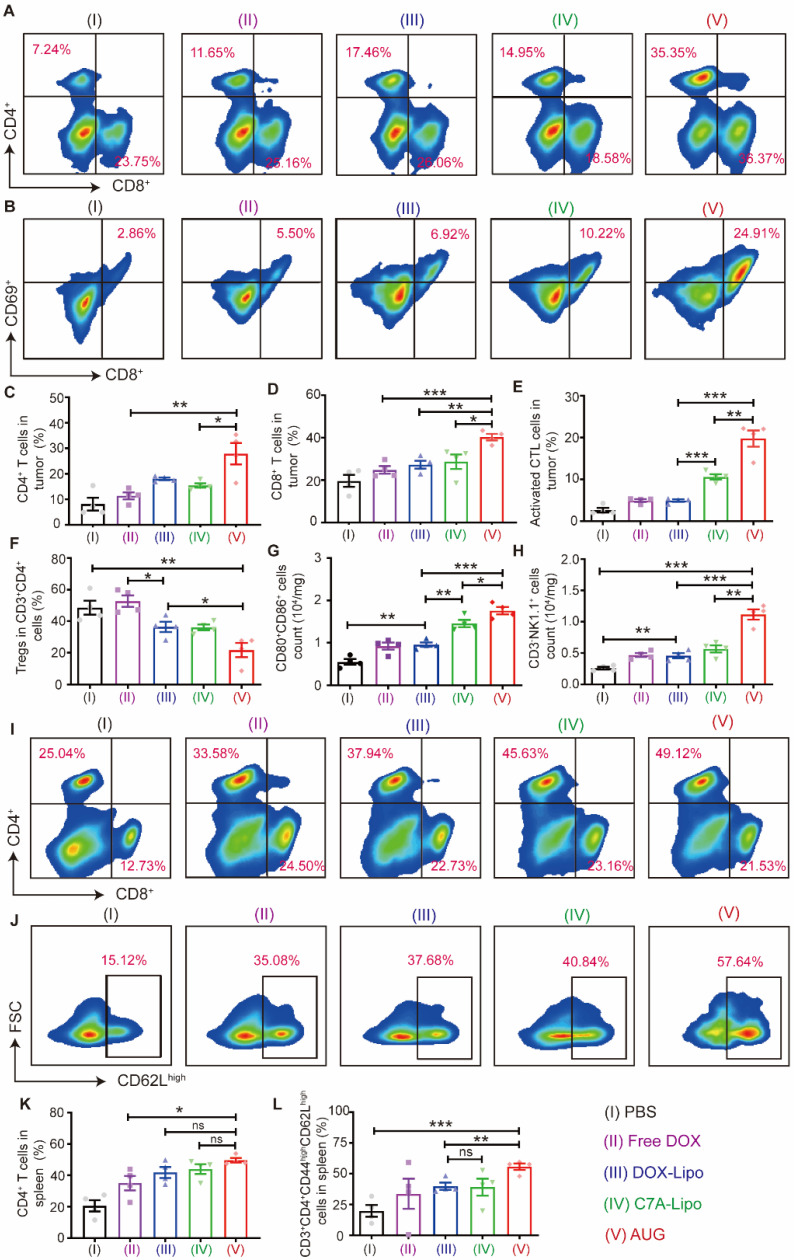
The immunologic mechanisms of neoadjuvant chemo-immunotherapy with AUG. (A) Representative flow cytometry charts of CD4^+^CD8^+^ T cells in tumors. (B) Representative flow cytometry charts of CTLs in tumors. (C) Relative quantitative of CD4^+^ T cells out of CD3^+^ T cells. (D) The proportion of CD8^+^/CD3^+^ T cells. (E) Relative quantitative of CTLs out of CD3^+^ T cells. (F) Percentage of Tregs in tumor. (G) Maturation of CD80^+^CD86^+^ DCs in tumor. (H) Activated NK cells (CD3^-^NK1.1^+^) in tumor. (I) Representative images of flow cytometric analysis of CD4^+^CD8^+^ T cells in spleen (gated on CD3^+^ T cells). (J) Representative images of flow cytometric analysis of CD62L^high^ cells in spleen (gated on CD3^+^CD8^+^CD44^high^ cells). (K) Relative quantitative of CD4^+^ T cells out of CD3^+^ T cells in spleen. (L) Percentage of memory T cells (CD3^+^CD4^+^CD44^high^CD62L^high^) in spleen. *P < 0.05, **P < 0.01, ***P < 0.001.

Encouraged by the remarkable therapeutic effect *in vivo*, the mechanism of AUG in antitumor immunity was further explored (Figure S15). As presented in Figures 5A, C and D, the administration of AUG significantly increased the proportion of CD4^+^ and CD8^+^ T cells within tumors compared to other treatment groups. Following this, the expression of the activation marker CD69 of T cells was assessed. The percentage of CD8^+^CD69^+^ cells within the CD3^+^ population was notably elevated by AUG treatment (19.8 ± 2.4%, gated on CD3^+^), compared to other groups (PBS: 2.6 ± 1.7%; free DOX: 4.9 ± 0.4%; DOX-Lipo: 4.9 ± 0.2%; C7A-Lipo: 10.6 ± 1.6%) (Figure 5B and E). Furthermore, the presence of infiltrating regulatory T cells (Treg), which can impede the function of CD8^+^ T cells, was significantly reduced in AUG-treated group (21.7 ± 5.9%) compared to the control groups (PBS: 48.5 ± 4.9%; free DOX: 52.7 ± 5.3%; DOX-Lipo: 36.4 ± 4.5%; C7A-Lipo: 36.1 ± 1.7%) (Figure 5F). Collectively, these findings suggest that AUG elicits an immune response in tumors, where adaptive immunity plays an important role.

We then detected the number of mature DCs in tumors with various treatments. As shown in Figure 5G, mature DCs of the AUG group increased to 1.8- and 1.3-fold compared with DOX-Lipo and C7A-Lipo groups, respectively. In addition, natural killer (NK) cells are innate lymphocytes with cytotoxic properties that eliminate tumor cells, and STING agonism has been found to enhance the antitumor activity of NK cells 38. A decrease in pH (pH 6.5) would lead to the breakage of hydrazone bonds and the subsequent release of C7A, which can rejuvenate NK cells. To confirm our hypothesis, the activation of NK cells (CD3^-^NK1.1^+^) was measured. The results depicted in Figure 5H demonstrated a significant increase in NK cell activation in tumors treated with AUG compared to other experimental groups. Specifically, the number of activation NK cells in the AUG group was 4.3-fold, 2.4-fold, 2.5-fold and 2.0-fold higher than the PBS, free DOX, DOX-Lipo and C7A-Lipo groups. These results indicate that AUG also could reactivate innate immunity response in the tumor microenvironment and favor suppressing tumor growth.

The spleen, being the largest immune organ in the body, is essential in the immune defense system through the synthesis of immune active substances like antibodies and lymphokines for tumor immunity. We therefore detected CD4^+^ and CD8^+^ T cells in the spleen after treatments using different formations (Figure S16). Our findings, illustrated in Figure 5I and K, indicated a significant increase in the proportion of CD4^+^ T cells in the spleen following AUG treatment (49.6 ± 3.7 %) compared to the PBS group (20.6 ± 6.9%) and DOX group (35.1 ± 10.3%). We further tested the T_CM_ (CD3^+^CD4^+^CD44^high^CD62L^high^) cells, and remarkedly increased of T_CM_ cells were observed in AUG group (55.7 ± 4.2%, gated on CD3^+^CD4^+^CD44^high^), compared with PBS (19.9 ± 8.5%) and free DOX group (33.7 ± 13.5%) (Figure 5J and L). These results suggest that AUG treatment enhances immune system activation and the initiation of durable immune protection. Moreover, the H&E staining images presented in Figure S17 indicate the absence of kidney injury, pulmonary toxicity, cardiac disruption, and inflammatory infiltrates in the spleen across all experimental groups. In addition, levels of TP, ALB, AST, ALP, CKMB, and UREA in all groups fell within normal ranges (Figure S18), indicating no significant toxicity. In addition, WBC, RBC, HGB and PLT in blood were within healthy ranges (Figure S19). These findings supported the security of AUG.

### Prevention of postsurgical tumor recurrence and metastasis by AUG combined with αPD-1

**Figure 6:**
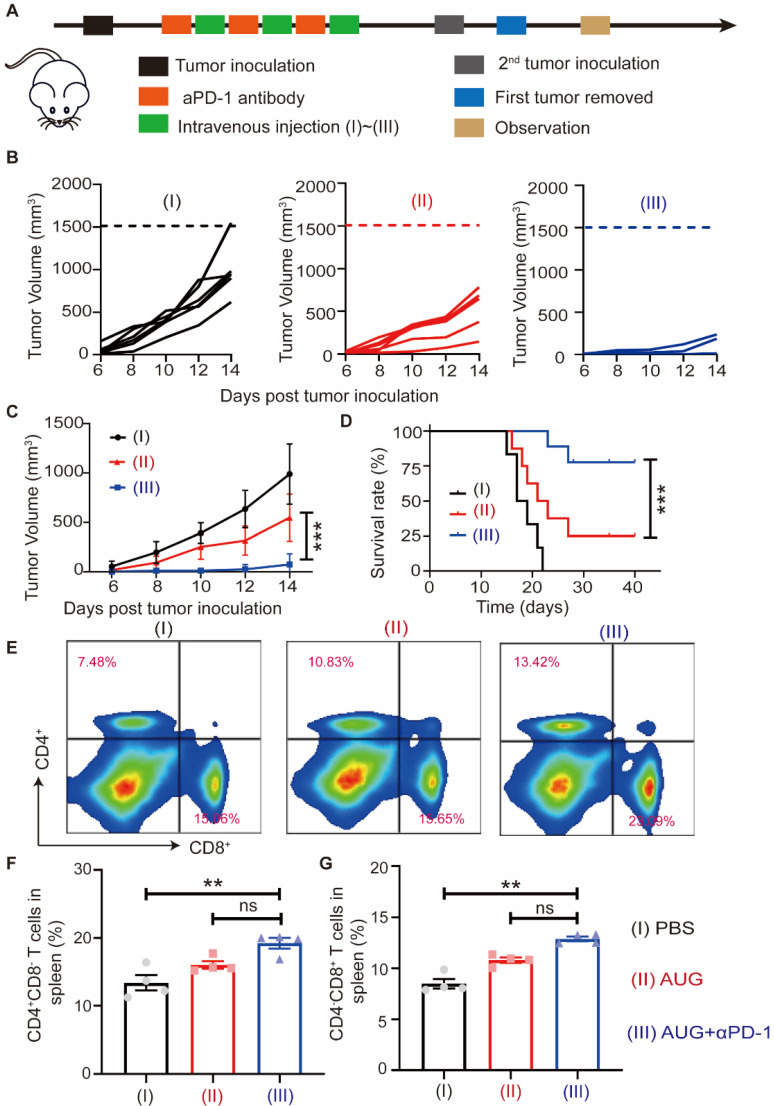
AUG can inhibit tumor metastasis after surgery as a neoadjuvant agent. (A) Schematic illustration of the schedule for assessing B16-F10 tumor metastases after surgery. (B) Tumor growth kinetics for each mouse. (C) Tumor growth curves of mice treated with PBS, AUG and AUG + αPD-1. (D) Survival curve of mice after treatment with PBS, AUG and AUG + αPD-1 groups. (E-G) Flow cytometry analysis and statistics of T cells in the spleen. *P < 0.05, **P < 0.01, ***P < 0.001.

Tumor recurrence and metastasis are major factors in mortality in cancer patients undergoing surgical intervention, and immune memory plays a vital role in reducing the risk of postoperative tumor recurrence and metastasis. To investigate the effect of AUG on the prevention of metastasis, we established a postoperative metastatic tumor model of B16-F10 following the protocol outlined in Figure 6A. The addition of PD-1 blockade (αPD-1) has been proved in a significant increase in partial T and B cells and correlated with patient survival 39. Our results showed that both AUG and AUG + αPD-1 significantly inhibited tumor metastasis in a preoperative setting and the antitumor efficacy of AUG + αPD-1 group reached as high as 92% (Figure 6B and C). Besides, in comparison with AUG, the survival time of mice in AUG + αPD-1 group was significantly extended (Figure 6D). In order to elucidate the mechanism by which tumor metastasis is inhibited following a combination of AUG and αPD-1, flow cytometry was utilized to analyze the helper T lymphocytes and CTLs in the spleen. The results demonstrated that the AUG + αPD-1 group exhibited significantly higher helper T lymphocytes and CTLs ratios compared to the PBS group (Figure 6E-G), indicating the successful initiation of systemic immune responses. Furthermore, no significant differences were observed between the AUG and AUG + αPD-1 groups in terms of the ratio of CD4^+^CD8^-^ and CD4^-^CD8^+^ T cells (Figure 6F and G). A probable explanation is that AUG-induced ICD and activated cGAS-STING pathways can effectively enhance antitumor immune responses against tumor metastasis.

**Figure 7:**
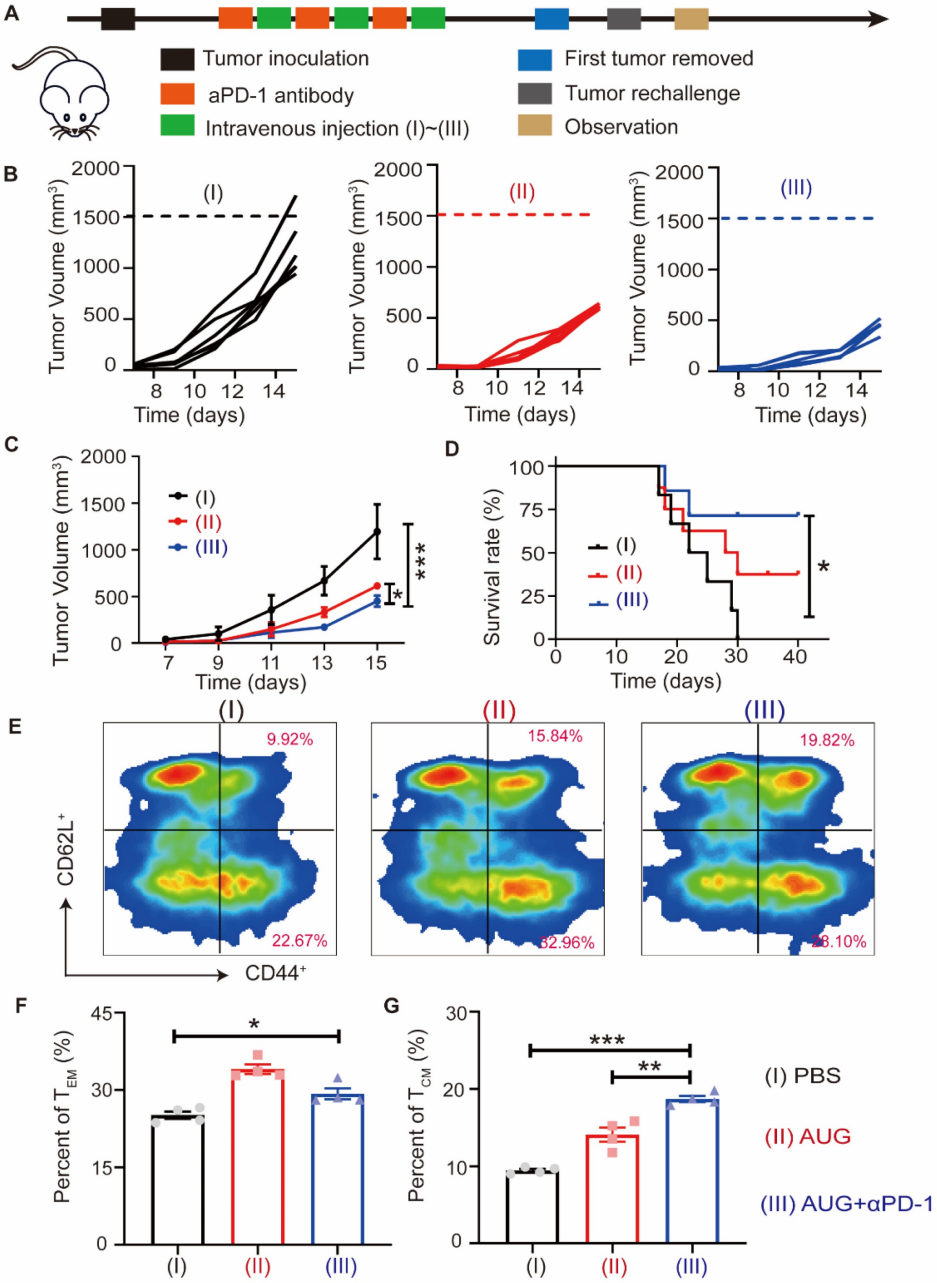
AUG can inhibit tumor recurrence after surgery as a neoadjuvant agent. (A) Schematic illustration of the schedule for evaluating postsurgical tumor recurrence. (B) Tumor growth kinetics for each mouse. (C) Tumor growth curves of mice treated with PBS, AUG and AUG + αPD-1. (D) Survival curve of mice after treatment with PBS, AUG and AUG + αPD-1 groups. (E-G) Flow cytometry analysis and statistics of T_CM_ and T_EM_ in the spleen. *P < 0.05, **P < 0.01, ***P < 0.001.

To model the recurrence of tumors following surgical treatment, B16-F10 cells were injected into the contralateral side of mice who had remained free of tumors for a period of 10 days (Figure 7A). It was found that the administration of the AUG + αPD-1 exerted a significant inhibitory effect (more than 62%) compared with PBS treated group (Figure 7B and C). We found that AUG treated alone exhibited similar antitumor efficacy as AUG + αPD-1 with slight differential. Furthermore, the AUG + αPD-1 treatment provided a strongest tumor inhibitory and leaded to an extended survival rate after tumor rechallenging (Figure 7D). To investigate the potential immunologic memory protection conferred by AUG, splenocytes were harvested the day prior to reintroduction of tumor cells for analysis of CD8^+^ T cells. Figures 7E-G and S20 demonstrated an increase in T_EM_ (CD3^+^CD8^+^CD44^high^CD62^low^) in the spleens of mice treated with AUG + αPD-1 compared to those in the PBS-treated mice. Furthermore, the proportion of T_CM_ (CD3^+^CD8^+^CD44^high^CD62^high^) was significantly greater in mice that received the combination of AUG and αPD-1 compared to that in AUG group and PBS group. Taken together, our results demonstrated that AUG plus αPD-1 could elicit the stronger immunological memory to effectively inhibit postsurgical recurrence of tumor and prolong the survival of mice.

## Conclusions

In this study, we constructed a hierarchical acidity-unlocked nanoSTING stimulant liposome as a neoadjuvant chemoimmunotherapy strategy that effectively prevented postoperative tumor recurrence and metastasis by activating both innate and adaptive anti-tumor immune responses prior to surgery, achieving durable therapeutic advantages. AUG exhibited excellent performance, such as being stable in the physiological environment, cascading the release of C7A and DOX in the tumor microenvironment to induce ICD and activate the STING pathway. As a result, AUG showed superior efficacy in inhibiting the growth of preoperative and postoperative melanoma. Therefore, AUG has the potential to pave the way for the advancement of neoadjuvant chemoimmunotherapy as a durable anti-tumor treatment.

## Supplementary Material

Supplementary figures and tables.
