# Supplementary material for: A hierarchically acidity-unlocking nanoSTING stimulant enables cascaded STING activation for potent innate and adaptive antitumor immunity
Source: Theranostics. 2024 Sep 16;14(15):5984–98. doi: 10.7150/thno.98272 (PMC11426243; doi:10.7150/thno.98272)
Supplement: Supplementary file 1 — Supplementary figures and tables. [file thnov14p5984s1.pdf]

A hierarchically acidity-unlocking nanoSTING stimulant enables cascaded STING activation for potent innate and adaptive antitumor immunity

Shunyao Zhu[1,+], Tao He[1,+], Yan Wang[1], Yushan Ma[2,3], Wenmei Li[4], Songlin Gong[1], Yanghui Zhu[1], Xiangwei Wang[1], Xu Xu[5], Qinjie Wu[1], Changyang Gong[1,*], Yanjie You[2,3,*]

[1] Department of Biotherapy, Cancer Center and State Key Laboratory of Biotherapy, West China Hospital, Sichuan University, Chengdu, 610041, China

[2] Department of Gastroenterology, the Third Clinical Medical College of Ningxia Medical University, Yinchuan, 750002, China

[3] Department of Gastroenterology, People's Hospital of Ningxia Hui Autonomous Region, Yinchuan, 750002, China

[4] Department of Clinical Laboratory Medicine, People's Hospital of Ningxia Hui Autonomous Region, Ningxia Medical University, Ningxia Hui Autonomous Region, Yinchuan, 750002, China

[5] School of Pharmacy, Henan University, Kaifeng, 475004, China

* To whom correspondence should be addressed (C Gong and Y You), E-mail: chygong14@163.com and youyanjie@163.com.

[+] These authors contributed equally to this work.

Table S1 List of antibodies applied to flow cytometric analysis.

Antibody	Clone	Fluorophore	Source
CD3	17A2	PE-cy7	Biolegend
CD3	17A2	APC/Cyanine5	Biolegend
CD4	GK1.5	PE	Biolegend
CD45	QA17A26	APC	Biolegend
CD8a	53-6.7	FITC	Biolegend
CD8a	53-6.7	PE/Cyanine5	Biolegend
CD80	16-10A1	APC	Biolegend
CD86	GL-1	PE-cy7	Biolegend
CD69	H1.2F3	PE	Biolegend
CD25	3C7	FITC	Biolegend
CD11b	M1/70	FITC	Biolegend
NK1.1	S17016D	PE	Biolegend
CD44	IM7	FITC	Biolegend
CD62L	MEL-14	APC	Biolegend
Foxp3	FJK-16s	APC	eBioscience
CD16/32 (Fc Block)	93	--	eBioscience
γH2AX	S139	--	Abcam
dsDNA	J2	--	Cell Signaling Technology
STING	Ser365	--	Cell Signaling Technology
p-STING	Ser366	--	Cell Signaling Technology
IRF3	D83B9	--	Cell Signaling Technology
p-IRF3	SER396	--	Cell Signaling

			Technology
TBK1	D1B4	--	Cell Signaling Technology
p-TBK1	Ser172	--	Cell Signaling Technology
GAPDH	D4C6R	--	Cell Signaling Technology

Table S2 The long-term stability of AUG at 4 ℃ (n = 3).

	Size (nm)	Polymer dispersity index (PDI)	Zeta potential (mV)	Doxorubicin (%)
0 Day	152.4 ± 1.6	0.218 ± 0.023	-10.42 ± 1.07	100.0 ± 0.3
7 Day	154.7 ± 2.9	0.168 ± 0.019	-4.51 ± 0.16	99.7 ± 0.9
14 Day	154.1 ± 3.0	0.144 ± 0.018	-6.49 ± 0.43	98.6 ± 1.3
21 Day	154.7 ± 0.4	0.167 ± 0.009	-5.17 ± 1.24	99.2 ± 0.8
28 Day	155.3 ± 1.0	0.161 ± 0.008	-4.16 ± 0.76	99.4 ± 1.0

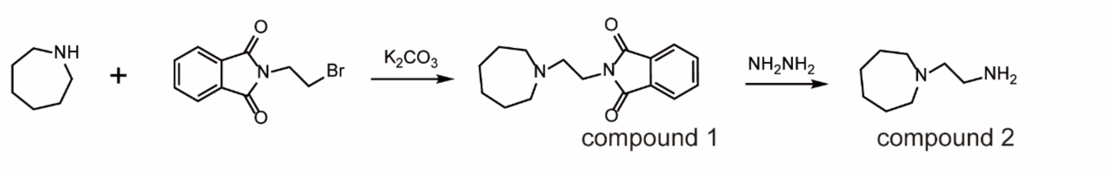

Figure S1. The synthesis routes of C7A-NH_2 (compound 2).

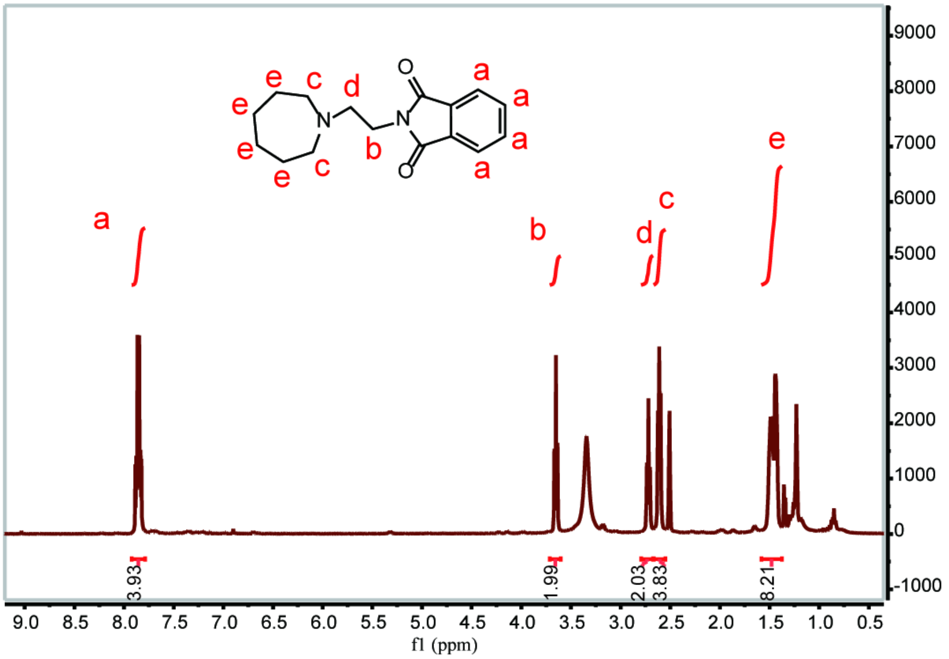

Figure S2. ^{1}H NMR spectrums of compound 1.

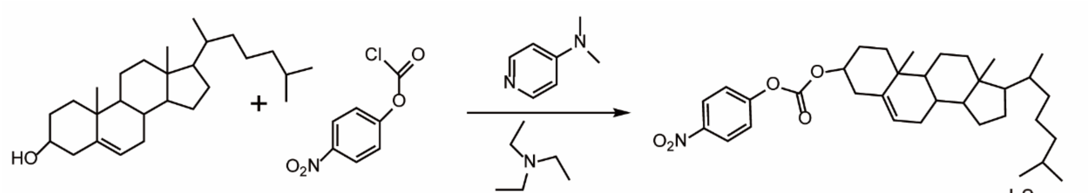

compound 3

Figure S3. The synthesis routes of CHOL-COOR.

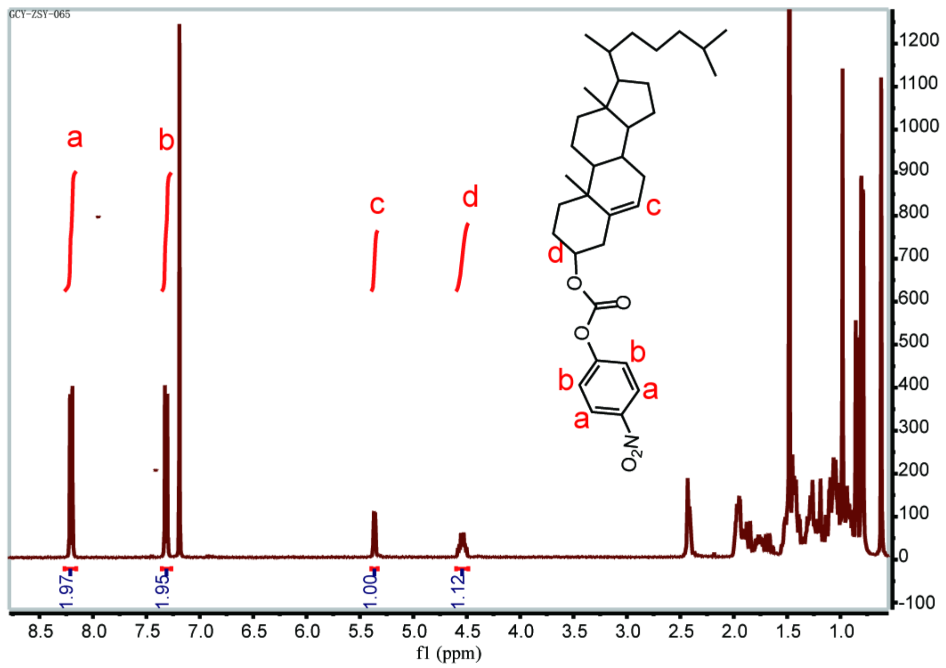

Figure S4. ^{1}H NMR spectrums of CHOL-COOR

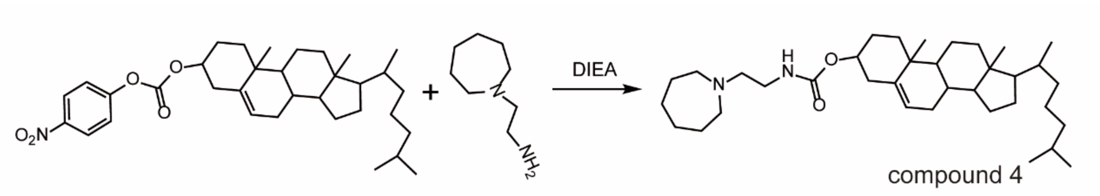

Figure S5. The synthesis routes of CHOL-C7A.

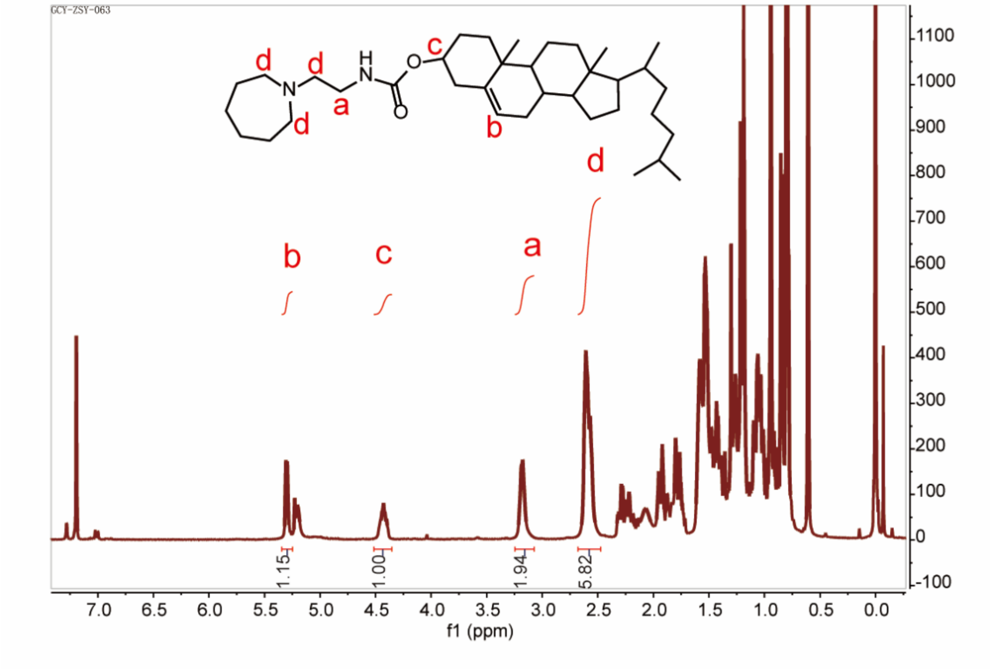

Figure S6. ^{1}H NMR spectrums of compound CHOL-C7A.

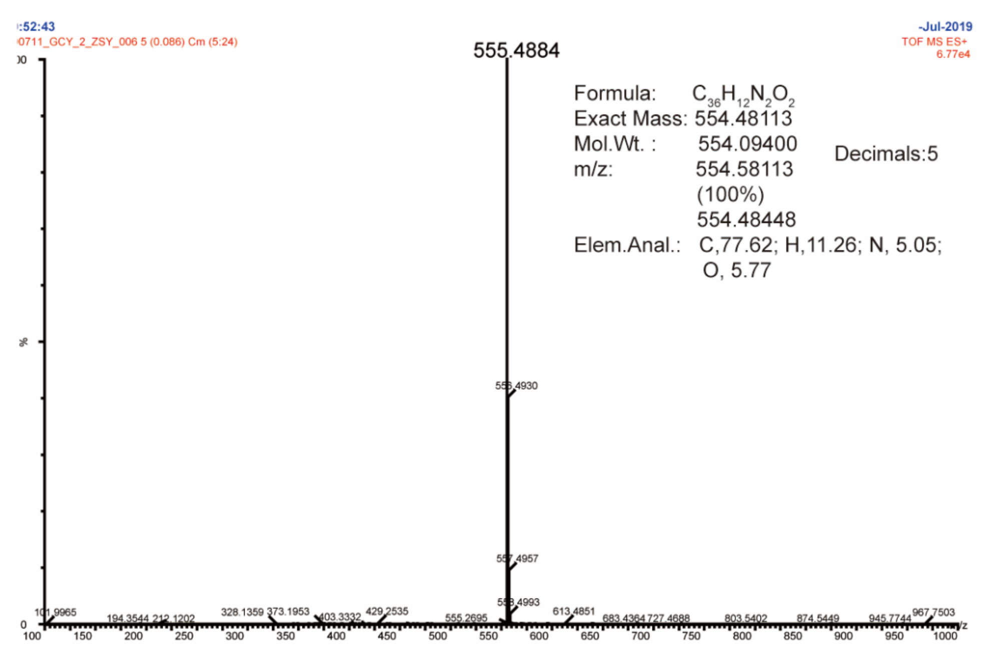

Figure S7. MS spectra of CHOL-C7A.

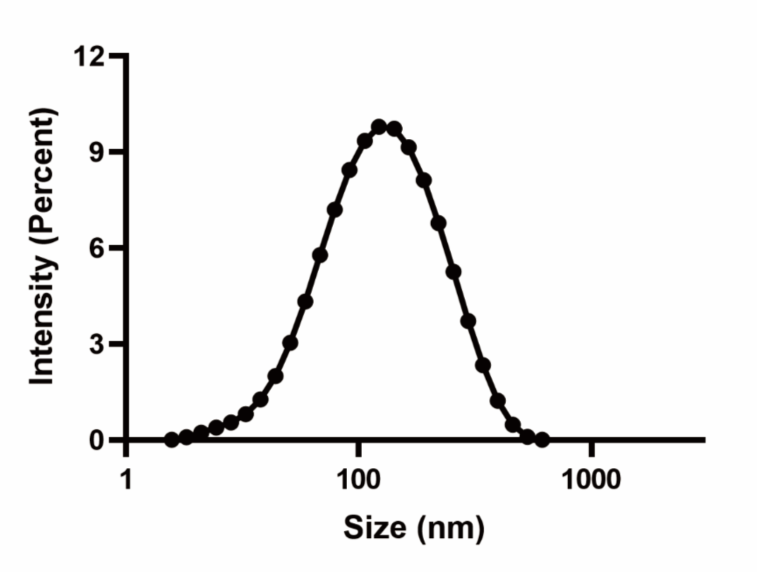

Figure S8. Size distribution of C7A-Lipo at pH 7.4.

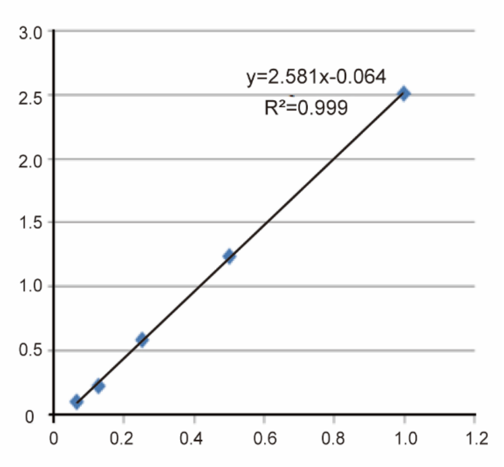

Figure S9. The standard curve was prepared by CHOL-C7A.

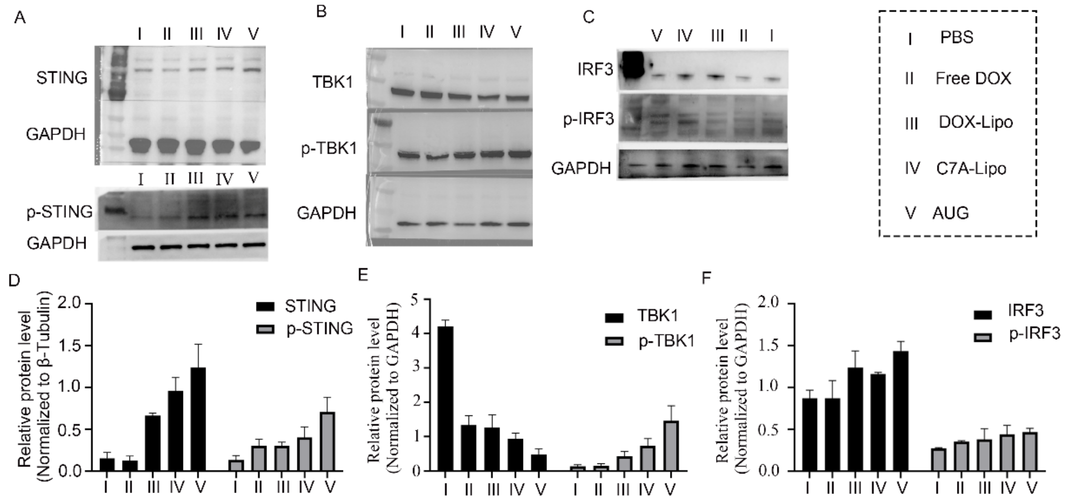

Figure S10. Representative gel image and quantitative statistics of (A) STING and p-STING; (B) TBK1 and p-TBK1. (C) IRF3 and p-IRF3 proteins expression in B16-F10 cells within different treatments.

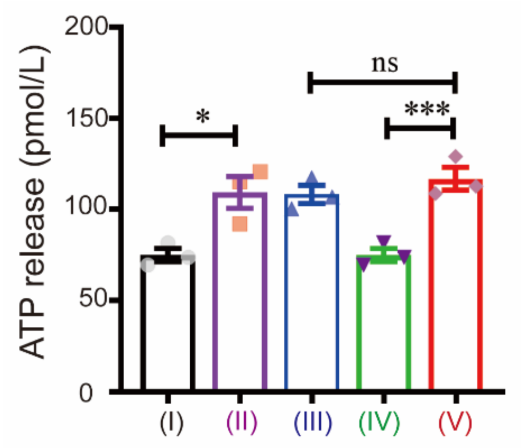

Figure S11. Quantification of ATP released from B16-F10 cell induced by DOX (n = 3). (I) PBS; (II) Free DOX; (III) DOX-Lipo; (IV) C7A-Lipo and (V) AUG. Data are means ± SD. *$P < 0.05$, **$P < 0.01$, ***$P < 0.001$ determined by Student's t-test.

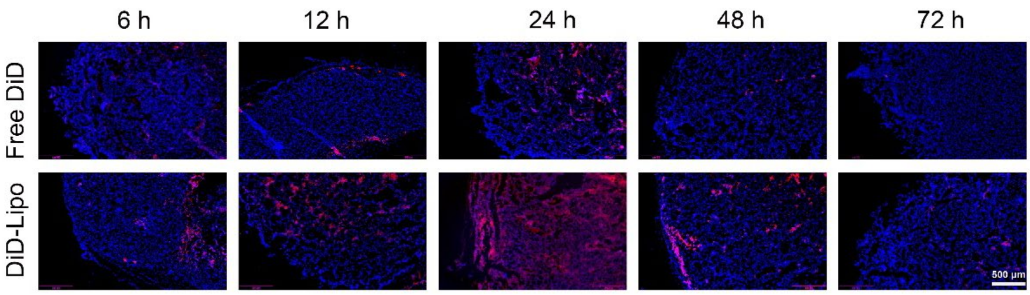

Figure S12. Representative fluorescence images depicting the infiltration and subsequent elimination of the therapeutic agent AUG within tumor tissues, with AUG being fluorescently labeled with DiD for enhanced visualization. Scale bar: 500 μm.

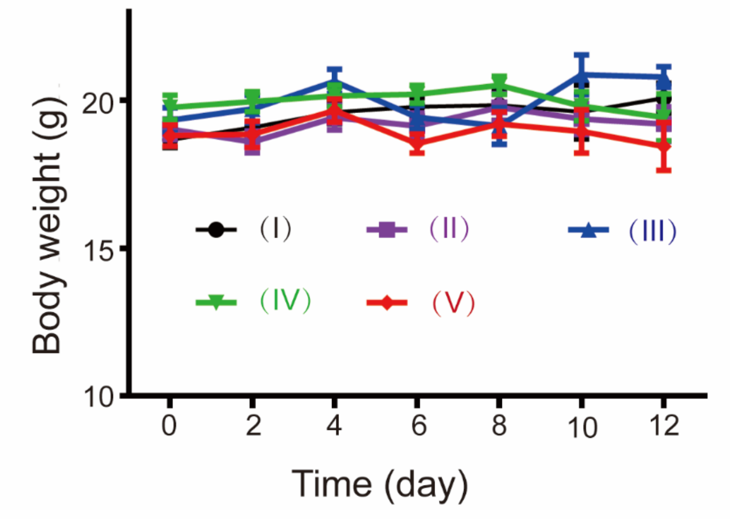

Figure S13. Changes of body weight during the treatment (n = 3). (I) PBS; (II) Free DOX; (III) DOX-Lipo; (IV) C7A-Lipo and (V) AUG.

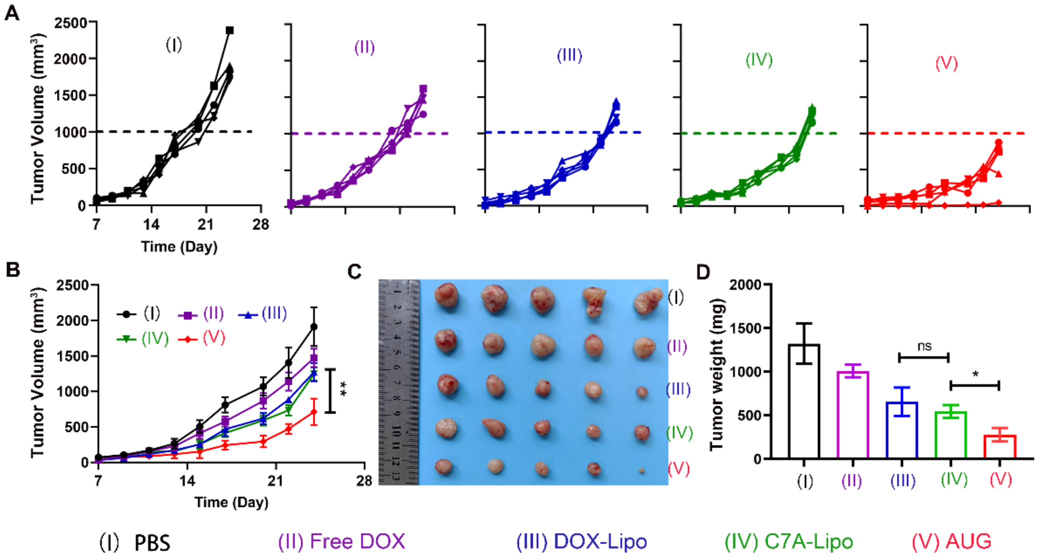

Figure S14: The antitumor effect of AUG in MC38 tumor. (A) Tumor growth curves for each mouse. (B) Total tumor growth curves of mice in different groups post-treatment. (C and D) Photographs of dissected tumors (C) and average tumor weights (D) at the end of the treatment. Ns = non-significant, *$P < 0.05$, **$P < 0.01$, ***$P < 0.001$.

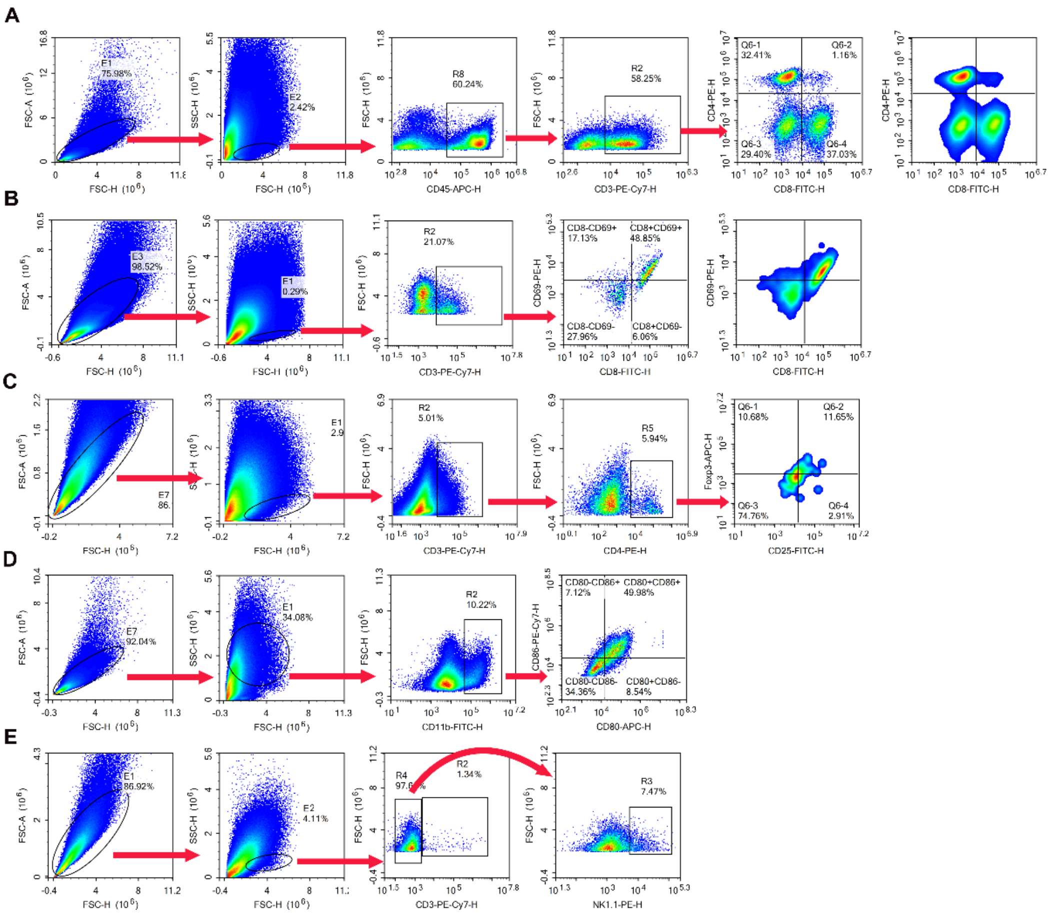

Figure S15: The gating strategy for the tumor microenvironment. (A) CD4$^+$ and CD8$^+$ T cells in tumor microenvironment. (B) CD8$^+$CD69$^+$ cells in CD3$^+$ T cells. (C) The proportion of Treg cells in tumor microenvironment. (D and E) Calculate the number of mature DCs (D) and NK cells (E) in the tumor microenvironment. The percentages indicated correspond to the relative frequency of cells within the respective parent gate, providing a clear representation of the cellular composition.

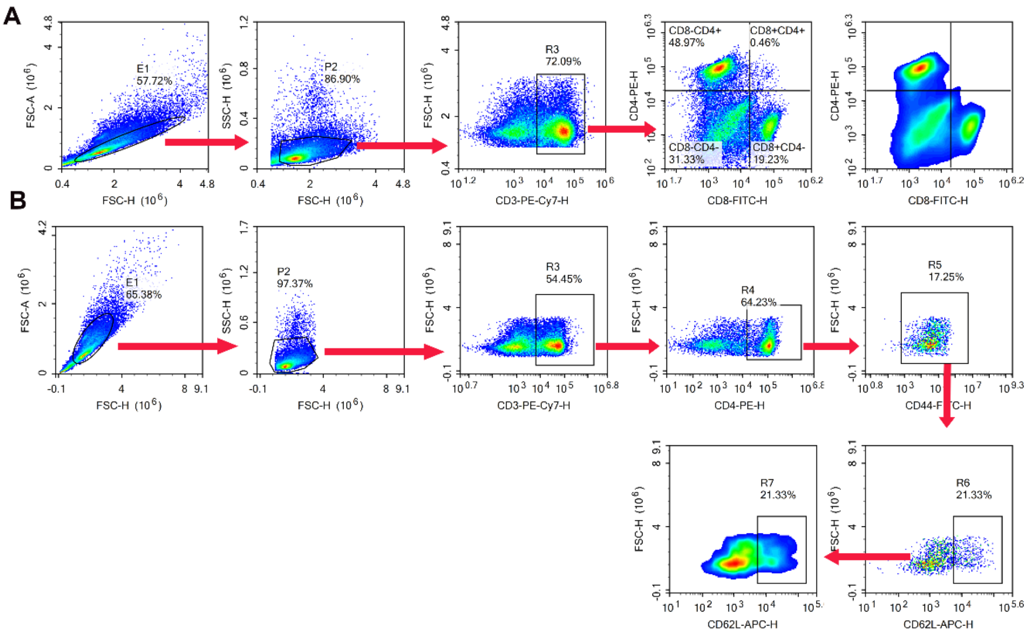

Figure S16. The gating strategy for manual analysis to sequentially identify the proportion of $CD4^{+}/CD8^{+}$ (A) and $CD62L^{+}$ (B) expressed in the spleen. Note: the percentages shown represent the frequency of cells relative to the parent gate.

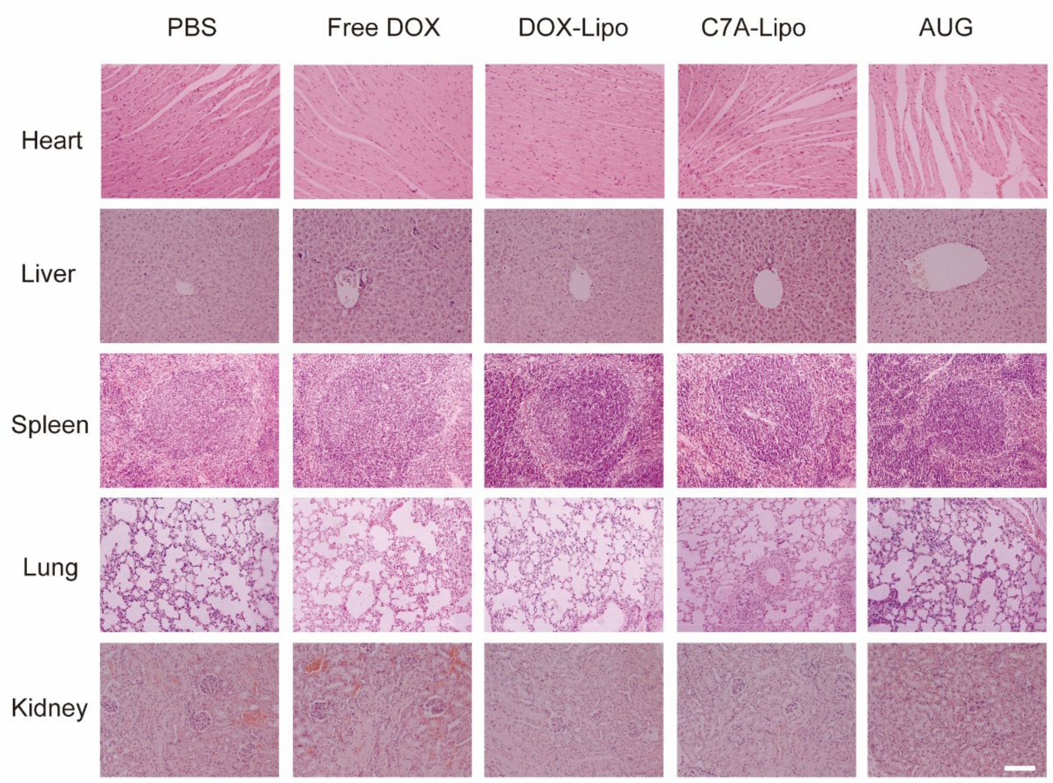

Figure S17. H&E staining of tissues including heart, liver, spleen, lung and kidney from C57BL/6 mice after various formulation treatment. The scale bar is 100 μm.

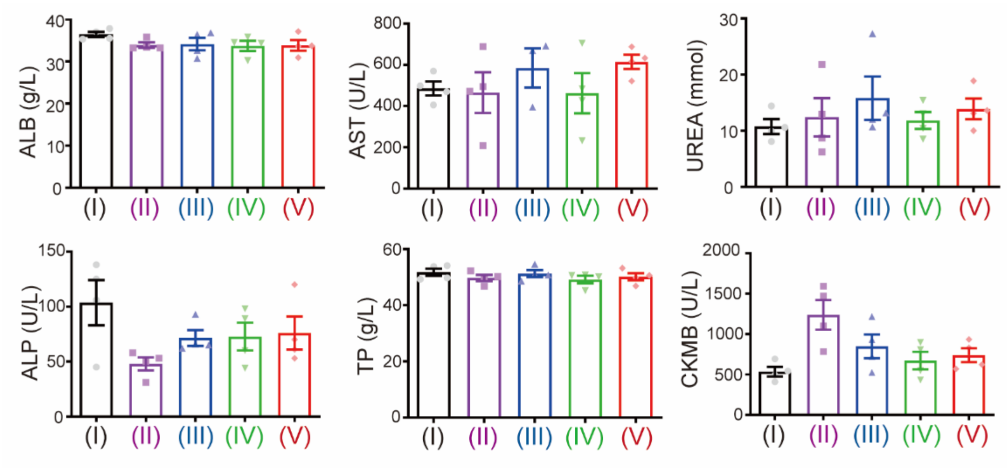

Figure S18. Serum chemistry levels in mice, including ALB, AST, UREA, ALP, TP and CKMB. (I) PBS; (II) Free DOX; (III) DOX-Lipo; (IV) C7A-Lipo and (V) AUG (n = 4). Data are means ± SD. *P < 0.05, **P < 0.01, ***P < 0.001 determined by Student's t-test.

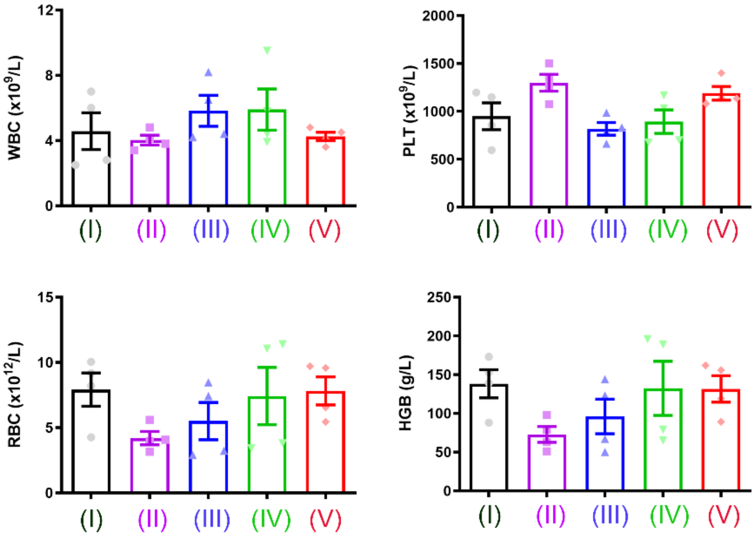

Figure S19. Blood routine levels in mice after treatment, including WBC, RBC, HGB and PLT. (I) PBS; (II) Free DOX; (III) DOX-Lipo; (IV) C7A-Lipo and (V) AUG (n = 4). Data are means ± SD. *$P < 0.05$, **$P < 0.01$, ***$P < 0.001$ determined by Student's t-test.

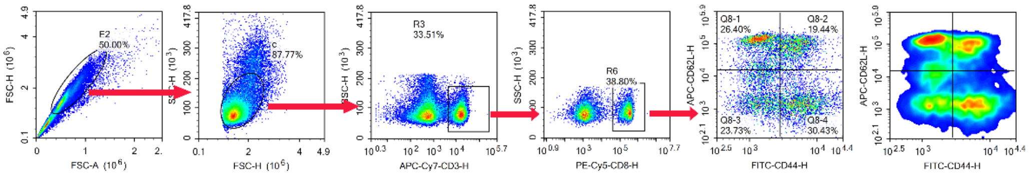

Figure S20. The gating strategy for manual analysis to sequentially identify the proportion of T_{CM} and T_{EM} cells in the spleen. Note: the percentages shown represent the frequency of cells relative to the parent gate.
